# Understanding factors influencing the use of specialized outdoor fitness equipment among older adults in Australia

**DOI:** 10.1093/heapro/daae160

**Published:** 2024-11-26

**Authors:** Chahana Paudel, Anna Timperio, Venurs Loh, Jo Salmon, Benedicte Deforche, Jenny Veitch

**Affiliations:** Institute for Physical Activity and Nutrition (IPAN), School of Exercise and Nutrition Sciences, Deakin University, 221 Burwood Highway, Burwood, VIC 3125, Australia; Institute for Physical Activity and Nutrition (IPAN), School of Exercise and Nutrition Sciences, Deakin University, 221 Burwood Highway, Burwood, VIC 3125, Australia; Institute for Physical Activity and Nutrition (IPAN), School of Exercise and Nutrition Sciences, Deakin University, 221 Burwood Highway, Burwood, VIC 3125, Australia; Institute for Physical Activity and Nutrition (IPAN), School of Exercise and Nutrition Sciences, Deakin University, 221 Burwood Highway, Burwood, VIC 3125, Australia; Faculty of Medicine and Health Sciences, Department of Public Health and Primary Care, Ghent University, C. Heymanslaan 10, 9000 Ghent, Belgium; Faculty of Physical Education and Physiotherapy, Department of Movement and Sport Sciences, Vrije Universiteit Brussel, Pleinlaan 2, 1050 Brussel, Belgium; Institute for Physical Activity and Nutrition (IPAN), School of Exercise and Nutrition Sciences, Deakin University, 221 Burwood Highway, Burwood, VIC 3125, Australia

**Keywords:** active ageing, outdoor gym, senior exercise park, green space, physical activity

## Abstract

This study assessed the use of newly installed outdoor fitness equipment (OFE) designed for older adults in two urban parks in Melbourne, Australia, and explored barriers and facilitators to its use among older adults. Direct observations were conducted using System for Observing Play and Recreation in Communities (SOPARC) to assess OFE usage by older adults (≥60 years) at two time points: T1 (November 2021) and T2 (October 2022). Additionally, 140 older adults (60–86 years, 59.3% female) were interviewed at T2 to explore their perceptions of OFE. Across both parks and timepoints, OFE usage by older adults was minimal. At T1, only 0.7% of older park visitors at Park A were observed using the OFE, while no visitors at Park B were observed using it. At T2, 2.8% and 0.7% of older adults at Parks A and B, respectively, were observed using the OFE. Interviews revealed various barriers to OFE usage, including personal factors (health concerns and time constraints), lack of knowledge about the equipment, perceptions about equipment suitability, and crowding by children. Facilitators for enhancing use of OFE included promotional efforts, instructional classes, improved equipment design and encouraging social engagement. Given the minimal usage of OFE by older adults identified in this study, addressing barriers and leveraging facilitators are essential to maximize the return on public investment, promote active ageing and foster improved overall well-being among older adults.

**Contribution to Health Promotion**

- Despite the installation of OFE specifically designed for older adults, minimal usage by older adults was observed in this study, indicating that simply providing the equipment is insufficient.
- Multiple factors, such as promotional efforts, programming and improved design, can facilitate increased utilization.
- Diversifying the types of OFE available to cater to varying fitness levels and preferences among older adults, including equipment that can be adjusted for different intensity levels and a mix of strength training, balance and flexibility exercises, is recommended.

## BACKGROUND

The global population is undergoing a notable shift towards ageing with the number of older adults aged 60 years and older projected to reach 2.1 billion by 2050. This represents a significant increase from 1 billion older adults in 2020 (United Nations, 2019). In Australia, an estimated 4.2 million or 16% of the total population were aged 65 and older in 2020. This has increased from 8% of the total population in 1970 and is projected to grow to 21% by 2066 (Australian Institute of Health and Welfare, 2021). An ageing population usually has an increased risk of chronic diseases and disability resulting in decrements in health and quality of life (He *et al*., 2016). Promoting physical activity among older adults can play a crucial role in mitigating the development of various health conditions, improving independence and quality of life, and reducing the healthcare burden associated with an ageing population (Manini and Pahor, 2009).

Parks provide numerous benefits to physical, mental and social well-being by enabling physical activity, social connections and interactions with nature (Bedimo-Rung *et al*., 2005; Hartig *et al*., 2014; Douglas *et al*., 2017). In recent years, there has been a growing global interest in installing outdoor fitness equipment (OFE) in public parks to provide accessible and cost-effective opportunities for physical activity (Lee *et al*., 2018; Jansson *et al*., 2019; Veitch *et al*., 2021). OFE comprises stationary exercise stations placed in an outdoor setting, offering individuals opportunities to engage in various intensities of physical activity, flexibility, balance and muscle strengthening exercises (Scott *et al*., 2014; Lee *et al*., 2018). A qualitative Australian study found older adults commonly identified OFE as a desirable park feature that could encourage their physical activity levels (Veitch *et al*., 2020). Additionally, OFE in parks has been shown to attract new park visitors, increase park visitation and encourage social interaction among older adults (Cohen *et al*., 2012; Chow, 2013; Levinger *et al*., 2021).

Despite the growing presence of OFE (Jansson *et al*., 2019), there remains a significant research gap understanding how older adults use and perceive this equipment. A qualitative study conducted in Hong Kong with 32 older adults found inefficient maintenance, a limited variety of OFE options and the absence of supporting amenities as barriers to using OFE by older adults (Lee and Ho, 2021). However, OFE remained an important supplementary exercise option for the participants, contributing to physical well-being, social interaction and mood enhancement (Lee and Ho, 2021). Another qualitative study conducted in Taiwan interviewed 55 older adults and found that participants considered OFE an enjoyable park addition and used it for health improvement, recreation and social interaction, but they also expressed concerns about availability, safety, maintenance, and equipment design and operation (Chow, 2013). Despite useful insights, these studies were conducted exclusively among users of OFE and thus omitted the perspectives of those who do not use OFE. Furthermore, previous research conducted in Taiwan noted that older adults constituted the primary user group for OFE accounting for almost half of total OFE users (Chow *et al*., 2017; Chow and Wu, 2019). In contrast, the utilization of OFE by older adults in Australia is low compared to the use by other age groups (Veitch *et al*., 2021; Jansson *et al*., 2022). In a study conducted in seven locations in regional areas in New South Wales, Australia, it was found that only 6.3% of the total OFE users were older adults (Jansson *et al*., 2022). In another study conducted in a park in Victoria, Australia, no older adults were observed using OFE (Veitch *et al*., 2021). This further underscores the importance of understanding how older Australians perceive OFE.

Only one study has examined older adults’ perceptions of OFE in Australia, and they found that health-related reasons were the primary motivator for use of OFE (Stride *et al*., 2017). This study, conducted among users and non-users of OFE, identified key enablers such as provision of shade, a diverse range of equipment and availability of instructional classes. The barriers to use of OFE included a general disinterest or dislike of equipment, concerns about crowded equipment due to children, and time constraints (Stride *et al*., 2017). It is also worth noting that existing studies primarily examined OFE that mimicked ‘gym-type’ machines, focusing mainly on aerobic and strength training. Limited evidence exists on the perceptions of older adults regarding OFE specially designed for older adults to target balance, mobility, functional movement and range of motion.

This study aims to explore the usage of specialized OFE recently installed in two parks in Melbourne, Australia, and to understand the barriers and facilitators influencing the use or non-use of OFE among older adults.

## METHODS

This study is a part of a larger study conducted to examine the impact of park improvements at two parks in Melbourne, Australia, on park visitation and park-based physical activity (Paudel *et al*., 2024). Park improvements included the installation of OFE designed for older adults (details described below) and were completed by June 2021. Direct observations were conducted at both parks to assess the use of OFE by older adults at two time points after the improvements were made: T1 (November 2021) and T2 (October 2022). One-on-one intercept interviews with older park users (aged 60 years and above) were conducted during T2 to understand their perceptions of the newly installed OFE in the two parks. Data collection occurred on days with no forecast of rain. The mean daily maximum temperature at T1 was 18.7°C (range: 17.3–20.2°C), and at T2 was 19.3°C (range: 16.1–21.5°C) (data obtained from the Bureau of Meteorology). During T1 data collection, multiple restrictions were enforced in Melbourne to mitigate the spread of COVID-19. These measures included limitations on indoor gatherings at residences and venues such as cafes and gyms, adherence to physical distancing guidelines and restrictions on the size of outdoor groups, but no restriction of park visitation and use of OFE (Victoria State Government, 2020; Premier of Victoria, 2021). Ethical approval for this study was provided by the Deakin University Human Ethics Advisory Group (HEAG-H 117_2020).

### Study site

This study was conducted in two parks, situated within Bayside City Council in Melbourne, Australia. Park A is located approximately 15.5 km southeast of Melbourne CBD with a decile score of 9/10 for the Index of Relative Socio-economic Disadvantage (IRSD) at the suburb level, indicating a relatively higher socio-economic advantage. Park B is located about 11 km southeast of Melbourne’s CBD and has a decile score of 10/10 for the IRSD at suburb level. Both parks installed OFE, also known as ‘Senior Exercise Park’ (Lappset Group), especially designed for older adults. The OFE offers a diverse range of equipment, each serving a specific function or movement important for older adults (Levinger *et al*., 2022). The details about specific equipment and surrounding amenities in each park are provided below.

#### Park A

**Fig. 1:**
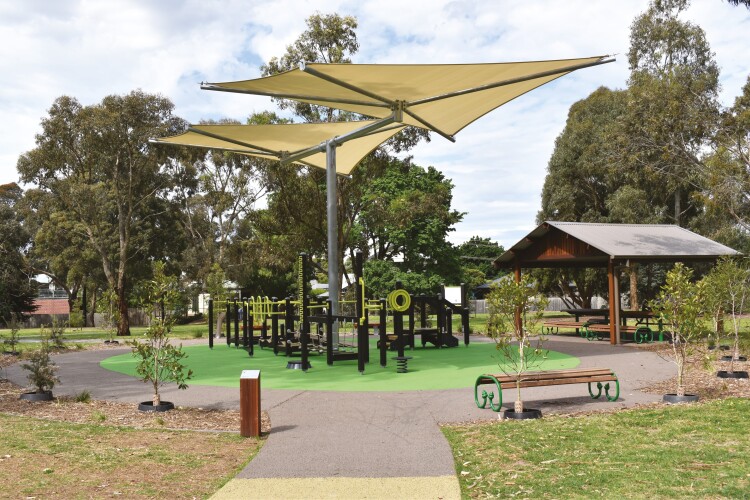
OFE in Park A.

The OFE in Park A (see Figure 1) has a 2.1 m wide circular level concrete pathway that surrounds the equipment and connects to the playground area (~50 m away) and a circuit walking path around the park. The OFE is installed on a rubber soft-fall surface with shade sails, an accessible drinking water fountain, a picnic shelter with table and seats, and additional seating around the equipment. It includes equipment designed to target balance, mobility, functional movement, flexibility and range of motion, such as walking balance beams, steps, pull-up/push-up bars, finger stairs and calf raise, hand roll, shoulder arches, gangway, walking ramp and net, balance stool and core twister. Instructional signage features the name and purpose of each piece of equipment and a quick response (QR) code allows users to access instructional videos demonstrating proper exercise techniques. The site also consists of signage indicating that the equipment is designed for older adults and that priority should be given to them. The Council also partnered with National Ageing Research Institute to train senior ‘champions’ in Park A to teach park users how to use the exercise equipment safely.

#### Park B

**Fig. 2:**
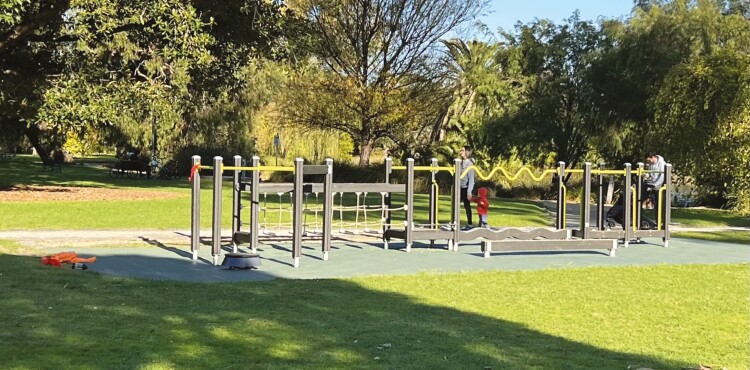
OFE in Park B.

The OFE in Park B (see Figure 2) is placed on a cushioned rubber/PVC surface and features a walking balance beam, gangway, pull-up and push-up bars, snake pipes, steps and a walking net. The OFE is adjacent to a walking path and near the children’s playground and a water fountain, picnic shelter with BBQ, table and seats are located nearby. During the data collection period, there were no instructional signage, or signage indicating that the equipment is specifically designed for older adults, and no shade over the equipment.

### Procedure and measurements

#### Direct observations

Observation scans were conducted using a modified version of the System for Observing Play and Recreation in Communities (SOPARC) (McKenzie *et al*., 2006). This observation method uses systematic scans to record the number of people present within designated target areas through momentary time sampling. At both parks, trained research assistants recorded the number of people who appeared to be older than 60 years actively using the OFE at that moment in time. Observations were completed at two timepoints T1 (November 2021) and T2 (October 2022). Data collection occurred every hour from 8:30 am to 5:30 pm (10 observation points/day) simultaneously at both parks over two weekdays and two weekend days. Observations were also conducted in other sections of the parks, however, only data representing total number of older adults present in the park was used in this study.

#### Interviews

At T2, a team of trained, clearly identifiable research assistants approached older park users, explained the study and all ethical considerations, and invited participation. Participants who could speak English and who met the age criteria were provided with a Plain Language Statement, and verbal consent was obtained before starting the interview. The interview guide examined topics including reasons for not using the OFE, factors that encouraged use of the OFE, and factors that would encourage them to use the OFE or use it more often. For descriptive purposes, participants were also asked to provide basic socio-demographic information such as age, sex, country of birth, and residential postcode, and some park use questions such as main activities when visiting the park, park accompaniment, and frequency of park visits and use of OFE in that park. The interviews lasted approximately 10 min and were conducted over two weekdays and two weekend days in between the observation scans. Responses were recorded on paper forms.

#### Data analysis

The total counts of older adults observed in each park and those using the OFE across all days were aggregated for each timepoint. Data from the interviews were manually transcribed into a digital format using Microsoft word and descriptive statistics were calculated for demographic characteristics in STATA. Transcribed data were reviewed by the lead author (C.P.) and verified for accuracy, ensuring that no information was omitted or altered during the transcription process. The dataset was then exported and analysed in NVivo 14 (QSR International, Melbourne, Australia) guided by conventional content analysis (Hsieh and Shannon, 2005). The process involved a recursive and iterative approach, allowing concepts to emerge from the data without preconceived notions. The coding (undertaken by C.P.) commenced using inductive methods to identify codes related to the research objective and were adopted throughout the analysis as new responses and content emerged. These codes were then grouped into themes and sub-themes, in consultation with other co-authors (J.V., V.L., A.T., J.S.). Interpretations were made within the context of the research objectives and the participants’ responses. Disagreements were discussed until consensus was reached.

## RESULTS

### Use of OFE by older adults

Table 1 shows the total number of observed older adults and those using OFE at the two parks at two time points. At T1, only 0.7% of older park visitors at Park A and no older park visitors at Park B were observed using the OFE. At T2, 2.8% of older park visitors at Park A and 0.7% at Park B were observed using the OFE.

**Table 1:** Counts of older adults observed visiting the two parks and using the OFE at each timepoint

Timepoint	Park A		Park B	
	Number of older adult park visitors (*n*)	Number of older adults using OFE *n* (%)	Number of older adult park visitors (*n*)	Number of older adults using OFE *n* (%)
T1	283	2 (0.7)	370	0 (0.0)
T2	362	10 (2.8)	292	2 (0.7)

### Interviews

Descriptive characteristics of the sample are presented in Table 2. A total of 69 interviews (60% female, mean age 69 years) were conducted at Park A, and 71 interviews (57% female, mean age 70.3 years) were conducted at Park B. Around 55% of participants in Park A and 79% in Park B lived in the same or neighbouring postcode of the park. Most participants at Park A visited the park with their grandchildren (57%), whereas at Park B, the majority visited with their partner or adult family members (47%). The main reason for visiting Park A was to take grandchildren to the playground, whereas at Park B the main activity was to go for a walk. Only 24 participants in Park A and 11 participants in Park B mentioned they had used the OFE in the respective parks and the majority of those who had used the OFE were infrequent users.

**Table 2:** Participant demographic characteristics from the interviews

	Park A (*N* = 69)	Park B (*N* = 71)
Sex *n* (%)		
Male	27 (39.1)	30 (42.3)
Female	42 (60.9)	41 (57.7)
Age, mean [SD], range	69.0 [5.6], 60–82	70.3 [7.4], 60–86
Country of birth, *n* (%)		
Australia	48 (69.6)	43 (60.6)
Other	21 (30.4)	27 (38.0)
Postcode of residence^a^, *n* (%)		
Local	38 (55.1)	56 (78.9)
Non-local	31 (44.9)	15 (21.1)
Usual frequency of visits to the park in past 3 months, *n* (%)		
≥ once per week	32 (46.4)	44 (62.0)
2–3 times per month	8 (11.6)	8 (11.3)
≤ once per month	23 (33.3)	18 (25.3)
Not visited the park before	6 (8.7)	1 (1.4)
Park companion^b^, *n* (%)		
Grandchildren	39 (56.5)	10 (14.1)
Partner or adult family members	18 (26.1)	33 (46.5)
Dog	10 (14.5)	15 (21.1)
Alone	10 (14.5)	11 (15.5)
Friends	4 (5.8)	11 (15.5)
Organized classes	2 (2.9)	—
Carer	1 (1.4)	1 (1.4)
Main activities when visiting the park^b^, *n* (%)		
Take grandchildren to playground	36 (52.2)	9 (12.7)
Walk the dog	13 (18.8)	18 (25.4)
Walk	8 (11.6)	31 (43.7)
Other exercises	3 (4.3)	—
Party/celebration	3 (4.3)	—
Relax	3 (4.3)	12 (16.9)
Socialize	2 (2.9)	3 (4.2)
Café/coffee	—	4 (5.6)
Fresh air/sunshine	—	5 (7.0)
Other	3 (4.3)	7 (9.9)
Frequency of OFE use in that park, *n* (%)		
≥ once per week	4 (5.8)	2 (2.8)
2–3 times per month	2 (2.9)	—
≤ once per month	17 (24.6)	9 (12.7)
Have not used OFE in that park	46 (66.7)	60 (84.5)

^a^Locals defined as participant living in same or neighbouring postcode to park.

^b^Multiple responses.

### Barriers and facilitators to use of OFE

Table 3 presents the themes and sub-themes related to facilitators and barriers for older adults’ use of OFE and includes the views of users and those who had not previously used the equipment. Under facilitators, four main themes emerged: equipment design and supporting amenities, guidance on usage/increased promotion, potential benefits and personal factors. Similarly, four main themes emerged for barriers: personal factors, lack of awareness and knowledge, suitability/perception of the equipment and crowding of children. Each of these themes has several sub-themes that elaborate further on the specific facilitators and barriers.

**Table 3:** Themes and sub-themes of barriers and facilitators to use of OFE that emerged from the interviews

	Themes	Sub-themes
Facilitators	Equipment design and supporting amenities	Availability of diverse range of equipment
		Supporting amenities such as benches, lights/shade
		Signage indicating children are not permitted to use
		Removing age-related labels
		Location relative to children’s playground
	Guidance on usage/increased promotion	Instructional or group classes
		Proper signage on how to use equipment
		Increased promotion of equipment
	Potential benefits	Perceived health benefits
		To pass time when supervising children
		Convenience and accessibility
	Personal factors	Frequent park visits
		Having more time available
		Advice from health professional
	Social engagement	Opportunity for social interaction
		Observing other people using the equipment
Barriers	Personal factors	Preference for alternative exercises
		Lack of interest
		Time/location constraints
		Health/safety concerns
		Discomfort with public exercise
	Lack of awareness and knowledge	Unfamiliarity with equipment
		Lack of awareness about target group
		Insufficient knowledge on how to use
	Suitability/perception of the equipment	Negative perception of equipment
		Perceptions about suitability of the equipment
	Crowding of children	Equipment used by children

### Facilitators to use of OFE

#### Equipment design and supporting amenities

The availability of amenities and equipment improvements were seen as facilitators by several participants. Participants from both parks expressed a desire for a wider range of equipment options to provide them with greater variety and cater to their individual preferences and fitness goals. Many participants in Park B also highlighted the need for shade over the equipment and additional amenities nearby.

*‘There should be different equipment and more variety—such as strength equipment for arms pulling’. 72 yrs, female, Park B*

*‘If it was tougher maybe I would use it. They should put tougher equipment around the current equipment or around walking paths so people could choose’. 72 yrs, male, Park A*

*‘There should be amenities such as benches, drinking water nearby, shade sail, more seatings, and shade’. 65 yrs, female, Park B*

Other factors that would encourage using the OFE included age-related labels, however, participants opinions regarding this were mixed. A few in Park A advocated for removal of signage designating the equipment for older adults, believing it should be accessible for children as well, while a few in Park B suggested adding signage to discourage children’s use and prevent overcrowding.

*‘There should be a signage saying it is not for kids. It needs to be secluded and cut off so that kids and dogs don’t come in’. 71 yrs, male, Park B*

*‘“Elderly exercise area”—I don’t like that it is a senior’s place—rename it. Get rid of the ageist aspect’. 80 yrs, male, Park A*

*‘Children like the area too, I’d advice to remove the age limit’. 64 yrs, female, Park A*

Similarly, preferences for the location of the OFE were mixed. Some participants in both parks preferred a degree of seclusion from children’s playgrounds, while some in Park A suggested having the equipment located closer to the children’s playground.

*‘Move away from playground—too clustered—too close to kids/ too exposed’. 64 yrs, male, Park B*

*‘It could be closer to the children’s playground so I could watch my grandson’. 77 yrs, male, Park A*

#### Guidance on usage/increased promotion

Guidance on usage and increased promotion of the OFE were seen as important factors for encouraging use by most participants. Participants expressed a strong desire for instructional or group classes that would provide them with the necessary knowledge and skills to effectively use the equipment. Furthermore, participants from both parks emphasized the value of having someone available to advise them on how to use the equipment to enhance their confidence and competence in utilizing the equipment.

*‘Something from council would be good, would definitely use if council ran exercises in equipment’. 70 yrs, female, Park A*

*‘Group classes to encourage use and understand how it works’. 68 yrs, female, Park B*

A few participants in Park A and a larger number of participants in Park B also emphasized the importance of signage that clearly illustrates how to use the equipment and safety guidelines. Additionally, participants highlighted the need for increased public promotion, awareness and communication about the OFE.

*‘I need explanation on what to do on it, proper signage on how to use it’. 72 yrs, female, Park B*

#### Perceived benefits

A few participants who had used the equipment found that the benefits they gained from using the OFE encouraged future use. They experienced improved balance, strength and overall health and appreciated the gentle impact on the body. Participants also expressed that using the OFE was enjoyable and provided a leisurely experience and a way to pass time, particularly when supervising children. Some participants mentioned the appeal of being able to exercise outdoors and enjoy the fresh air and appreciated the convenience and accessibility of the OFE.

*‘I come here with organised class- I have falling issues so I exercise here to improve health and to get extra strength’. 80 yrs, female, Park A*

*‘It’s fun to use, wobbling is fun’. 70 yrs, female, Park B*

#### Personal factors

Some participants suggested that frequent visits to the park or having more time available might serve as a facilitator to using the OFE. A small number of participants in Park B indicated that receiving advice from a health professional about using OFE could encourage them to use it.

*‘Just coming more often would encourage me to use it’. 70 yrs, male, Park A*

*‘I am usually busy minding children, if free from supervising children I would use it’. 66 yrs, male, Park B*

*‘I would use it if a doctor said so’. 77 yrs, male, Park B*

#### Social engagement

Participants highlighted that social aspects such as engaging in exercises with others, being accompanied by family members, or observing others exercising, would encourage them to use the equipment.

*‘A friend- I would love to use it with a friend’. 78 yrs, female, Park A*

*‘Other people exercising inspire me’. 70 yrs, female, Park A*

### Barriers to use of OFE

#### Personal factors

Many participants cited personal reasons as the primary factor behind reluctance to using the OFE. In particular, a preference for engaging in other exercises, such as walking or running was frequently mentioned by participants in both parks, while others also reported going to a gym. Several participants expressed a general lack of interest in using the equipment or did not perceive any personal benefits from doing so. A few participants from both parks also expressed a dislike for exercising in public.

*‘I go to gym regularly, I find this too simple’. 64 yrs, female, Park A*

*‘I don’t like exercising in public- I have my own fitness routine’. 63 yrs, female, Park B*

Time and location constraints were also cited as barriers. Some participants mentioned having limited time due to responsibilities such as supervising grandchildren or pets in parks and a few participants reported not living close enough to the park where the equipment was located. Participants from both parks expressed concerns related to existing health issues, which prevented them from engaging in vigorous physical activities and concerns about the potential strain or exacerbation of their health conditions and fear of falls if they were to use the OFE.

*‘I can’t because of health issues, I had back surgery’. 77 yrs, female, Park B*

*‘I’m not able to exercise on it’. (uses walking sticks), 67 yrs, female, Park A*

#### Lack of awareness and knowledge

Another prominent theme that emerged was unfamiliarity with the equipment and many participants from both parks were uncertain about the target group that the OFE was intended for with some (particularly in Park B) believing it was designed for children.

*‘Because its next to the playground, I didn’t realise it was for adults- looks just like kids’ equipment’. 71 yrs, female, Park B*

In addition, participants were also unaware about how to effectively use the equipment.

*‘I looked at it, wondered about it—seems difficult’. 79 yrs, female, Park B*

#### Suitability/perceptions of the equipment

A few participants expressed negative perceptions of the equipment, with some stating that the equipment was not challenging enough. These sentiments were observed in both parks, however, they were more prevalent among participants from Park B.

*‘There’s not good equipment here, I have used good equipment in other parks with proper machines’. 81 yrs, male, Park B*

*‘It’s not challenging enough, needs more variety’. 78 yrs, male, Park B*

Perceptions about the suitability of equipment were varied. Some participants felt they were not old enough or did not have mobility issues that necessitated the use of the equipment, whereas others felt that they were too old to use the OFE.

*‘… maybe if you have balance issues but not for me, maybe someone recovering from injury’. 68 yrs, male, Park A*

#### Crowding of children

A small number of participants from both parks mentioned that crowding of children around the equipment was a deterrent to its use. This was more prevalent in Park B compared to Park A.

*‘It’s too close to the kids’ playground so it is utilised by them’. 72 yrs, female, Park B*

## DISCUSSION

This study provided insights on use and perceptions of newly installed OFE among older adults in two parks in Melbourne, Australia. Findings from observational data showed that use of the OFE by older adults was minimal and the interview data highlighted barriers and facilitators of OFE use. Several facilitators were identified to promote engagement and regular use, including enhancing equipment design, providing guidance in the form of instructional classes, encouraging social engagement, providing information about OFE and highlighting the perceived benefits. Personal factors (such as preference for alternative exercises and health/safety concerns), lack of awareness and knowledge about OFE, perceptions about suitability of the equipment and crowding of children constituted barriers to use of the OFE. By leveraging these insights, strategies can be developed to promote usage of OFE and support active ageing and improved overall well-being among the older adult population.

Although self-reported usage of OFE during the interviews was higher than that recorded by direct observation, most individuals reported using the OFE less than once a month. This limited usage of OFE is consistent with past research conducted in Australia and Canada that reported OFE use by older adults to be 0% and 2.2% of the total older park visitors observed, respectively (Copeland *et al*., 2017; Veitch *et al*., 2021). Other studies conducted in the United States and Portugal have also found older adults to be among the least observed users of OFE compared to other age groups (Bettencourt and Neves, 2012; Cohen *et al*., 2012). The low usage of OFE among older adults, despite the equipment being specifically designed for this demographic, underscores that simply installing equipment is not enough to ensure regular use. This further highlights the importance of gaining insights from older adults regarding strategies that could encourage them to use the OFE so that these public investments have optimal use.

From the interview data, one of the key barriers identified was the lack of awareness and knowledge regarding the newly installed OFE. Many participants were unaware of the equipment and intended target group, perceiving the equipment as primarily designed for children, particularly in Park B. This indicates potential gaps in communication and promotion efforts. To ensure successful adoption of the OFE, it is important for local councils to implement targeted promotional campaigns. Past studies have emphasized the importance of marketing physical activity programs in conjunction with park improvements to encourage use and maintain increases in physical activity in the long term (Hunter *et al*., 2015; Cranney *et al*., 2016). For instance, an evaluation of the installation of an outdoor gym reported an increase in older adults’ outdoor gym usage due to continued promotion efforts targeting older adults before and after installation (Cranney *et al*., 2016).

Participants in the current study expressed a lack of familiarity with how to use the equipment and indicated the need for clearer instructions or guidance to facilitate usage among older adults. This is an important point as the equipment at Park A included QR codes linking to instructional videos and a physical instructional signage. However, participants at Park B, which lacked QR code and physical signage more frequently mentioned the need for clearer instructions. This suggests that simpler instructional information signage, as well as QR codes, are needed. These signs should state the purpose and intended user group, provide step-by-step instructions on proper equipment use, and highlight the potential health benefits for older adults. The importance of signage when designing outdoor fitness areas for older adults has also been emphasized in a recent narrative review (Marcos-Pardo *et al*., 2023). In addition, organizing instructional classes or group sessions may be key to raise awareness, provide practical instruction on how to use the equipment, and encourage older adults to engage with the equipment regularly and maintain their interest over time. Such interactions can also create a social support network, which has been cited as a key facilitator to using OFE in the present study as well as in a previous study (Chow, 2013). Future research should examine how psychosocial factors, including social support from exercise companions or community groups, influence OFE usage. Additionally, future studies should explore integration of mobile applications that offer personalized guidance, progress tracking, reminders and incentives to enhance use and adherence over time.

While both parks had minimal usage, it is likely that promotional efforts in Park A along with initiative to train champions may have contributed to slightly higher use of OFE compared to Park B. This suggests that even modest promotional activities can yield some improvements in OFE usage, underscoring the potential impact of more comprehensive and ongoing awareness and training programmes. Furthermore, involving local healthcare providers, to ‘prescribe’ the use of OFE could be an effective strategy to help raise awareness, provide credibility and motivate older adults to use the equipment as part of a healthy lifestyle. Such an approach has been successful in promoting physical activity among older adults in other contexts (Pfeiffer *et al*., 2001).

The participants in this study also had varying perceptions about the suitability and level of challenge offered by the OFE. These perceptions appeared to be influenced by their individual fitness levels, age and preconceived notions about exercise equipment. Many participants also expressed a general lack of interest, with some feeling that they saw no personal benefit in using the equipment. Some participants found the equipment to be too easy, not challenging enough for their fitness level and thought it to be more appropriate for someone older than them or for someone with mobility issues. This perception may have stemmed from the fact that the equipment was designed to prioritize balance/flexibility rather than focusing solely on strength training. Others believed they were either too old, not physically capable or lacked the necessary mobility to engage in the exercises, with health concerns or fear of injury or falls also raised. These findings suggest a general lack of understanding regarding the potential benefits of the equipment, as previous studies have shown that using this type of equipment can effectively minimize falls among older adults (Levinger *et al*., 2022). This further emphasizes the need to raise awareness about the equipment. In this study, many participants expressed a desire for diverse equipment especially for strength training. While the use of the current equipment offers significant advantages for older adults, it may be worthwhile to include a broader variety of equipment that can be adjusted to different intensity levels and cater to older and younger demographics with a wide range of fitness levels and abilities. In addition, specific training/group classes could be organized for subgroups of older adults with varying levels of capacity and needs.

The interview findings revealed that amenities and environmental factors were important to facilitate use of the OFE. Shade was highlighted as an important amenity, especially in Park B, which did not have shade over the equipment, which is consistent with findings from previous studies (Chow, 2013; Stride *et al*., 2017). Provision of additional benches, drinking water fountains and lighting were also cited as factors that may encourage use of OFE. It is worth noting, however, that there was minimal difference in observed usage between Parks A and B despite the presence of most of these supportive amenities in Park A. This suggests that while these amenities can make exercise more enjoyable, multiple factors may be at play in determining use of OFE by older adults.

Another important environmental factor to consider is the location of OFE in parks, mostly in relation to children’s playgrounds. Some older adults preferred using OFE while supervising their grandchildren and wanted it to be near the playground. Others viewed children as barriers, especially when the fitness area was close to the playground due to concerns about overcrowding and distractions. It may be that older adults find it difficult to focus primarily on their exercise routines with playgrounds nearby and crowding of children has also been cited as a barrier to using OFE in a previous study among older adults (Stride *et al*., 2017). To address this concern, participants suggested installing signage indicating that the OFE is intended for older adults, emphasizing its specific purpose and potentially discouraging children from playing on the equipment. However, some individuals advocated for removing age-related labels altogether, promoting inclusivity and allowing the equipment to be used by people of all ages, including children. This approach may also have the potential for intergenerational activities and foster shared enjoyment. Future research could investigate the impact of inclusive design strategies in OFE areas, assess the effectiveness of different approaches and their influence on user experiences, safety and engagement. Additionally, studies should explore how specific environmental characteristics, such as proximity to other recreational facilities, influence use of OFE.

## STRENGTHS AND LIMITATIONS

This research explored the use and perceptions of specialized OFE among older adults, a topic that has been relatively underexplored. The use of both direct observation and interviews with both users and non-users of OFE enriched the findings by capturing a wide range of opinions and experiences. This study also identified potential strategies to encourage equipment use, offering valuable insights that are important for the design and implementation of facilities that meet the needs and preferences of older adults, thereby encouraging greater participation and physical activity.

A limitation of this study is that it was conducted in only two parks in a relatively high socio-economic area of Melbourne, Australia; different levels of usage may be observed in parks in other areas where people may experience different facilitators and barriers. It is important for future studies to focus on lower SES areas to capture a broader range of experiences and needs, particularly as parks in lower SES may be less well resourced (Crawford *et al*., 2008; Cohen *et al*., 2016), and it is possible that many may not have OFE. While approximately 30% of the interviews were conducted among non-Australian-born participants, providing some cultural diversity, the findings may still lack generalizability to other locations or cultural contexts. For example, Asian countries such as Taiwan have shown to have a high prevalence of older adults using OFE compared to Australia (Chow *et al*., 2017). Future studies should consider including participants from diverse geographical locations to ensure broader applicability of the results. The usage patterns of different equipment types were not observed separately, so it is unclear which equipment was most popular. Additionally, it should be noted that the direct observations provided an indication of OFE use during specific days and times, and the outcomes may have been different if the observations had been completed on different days. Furthermore, park use may have been influenced by the COVID-19 restrictions in place during T1 data collection. Researchers estimated the age group of the park visitors; therefore, it is possible that some older adults who were perceived to be younger/older than 60 years were incorrectly included/excluded in direct observations or interviews, so use among older adults may have been under/overestimated. Finally, we acknowledge that ongoing or future promotional efforts may influence equipment usage and future studies should consider undertaking long-term follow-up and continuous evaluations to accurately track usage and perceptions of the OFE. These approaches could evaluate the sustained effects of OFE use on health and well-being of older adults over extended periods.

## CONCLUSION

This study investigated the use of OFE by older adults and identified several potential facilitators and barriers to overcome to enhance its usage. The observational data showed that use of the OFE by older adults was very low. The interview results highlighted a lack of awareness and knowledge about the equipment, concerns about crowding and personal factors such as age-related perceptions and health concerns as key barriers to usage. The findings underscore that provision of OFE alone is insufficient to promote regular use among older adults. To maximize the benefits of OFE, a multifaceted approach that includes incorporating targeted promotional campaigns, engagement from healthcare professionals, clear signages, instructional classes, a variety of equipment options and supporting amenities may be needed. This evidence can assist urban planners and policymakers to develop tailored interventions to ensure that OFE is accessible, inviting and suitable for older adults. The study findings underscore the importance of ongoing research in this area to refine and improve the design of OFE spaces, addressing evolving preferences and expectations of the ageing population.

## Data Availability

The datasets used and/or analysed during the current study are available from the corresponding author on reasonable request.
